# Clinical Implications of Azole Resistance in *Aspergillus fumigatus*, the Netherlands, 2007–2009

**DOI:** 10.3201/eid1710.110226

**Published:** 2011-10

**Authors:** Jan W.M. van der Linden, Eveline Snelders, Greetje A. Kampinga, Bart J.A. Rijnders, Eva Mattsson, Yvette J. Debets-Ossenkopp, Kuijper, Frank H. Van Tiel, Willem J.G. Melchers, Paul E. Verweij

**Affiliations:** Author affiliations: Radboud University Nijmegen Medical Centre, Nijmegen, the Netherlands (J.W.M. van der Linden, E. Snelders, W.J.G. Melchers, P.E. Verweij);; Groningen University Medical Centre, Groningen, the Netherlands (G.A. Kampinga);; Erasmus Medical Centre, Rotterdam, the Netherlands (B.J.A. Rijnders);; Utrecht University Medical Centre, Utrecht, the Netherlands (E. Mattsson);; Vrije University Medical Centre, Amsterdam, the Netherlands (Y.J. Debets-Ossenkopp);; Leiden University Medical Centre, Leiden, the Netherlands (E.J. Kuijper);; Maastricht University Medical Centre, Maastricht, the Netherlands (F.H. van Tiel)

**Keywords:** Aspergillosis, fungal infections, aspergillus, antifungal drug resistance, outcome assessment, prevalence, the Netherlands, research

## Medscape CME

Medscape, LLC is pleased to provide online continuing medical education (CME) for this journal article, allowing clinicians the opportunity to earn CME credit.

This activity has been planned and implemented in accordance with the Essential Areas and policies of the Accreditation Council for Continuing Medical Education through the joint sponsorship of Medscape, LLC and Emerging Infectious Diseases. Medscape, LLC is accredited by the ACCME to provide continuing medical education for physicians.

Medscape, LLC designates this Journal-based CME activity for a maximum of 1 *AMA PRA Category 1 Credit(s)*^TM^. Physicians should claim only the credit commensurate with the extent of their participation in the activity.

All other clinicians completing this activity will be issued a certificate of participation. To participate in this journal CME activity: (1) review the learning objectives and author disclosures; (2) study the education content; (3) take the post-test with a 70% minimum passing score and complete the evaluation at www.medscape.org/journal/eid; (4) view/print certificate.

Release date: September 22, 2011; Expiration date: September 22, 2012

## Learning Objectives

Upon completion of this activity, participants will be able to:

- Describe the prevalence of itraconazole resistance in clinical *A. fumigatus* isolates on the basis of a prospective, nationwide, multicenter surveillance study in the Netherlands
- Describe risk factors for development of itraconazole resistance in *A. fumigatus* isolates on the basis of that study
- Describe outcomes associated with development of itraconazole resistance in *A. fumigatus* isolates on the basis of that study

## MEDSCAPE CME EDITOR

**Nancy Mannikko, PhD, **Technical Writer/Editor, *Emerging Infectious Diseases. Disclosure: Nancy Mannikko, PhD, has disclosed no relevant financial relationships*.

## MEDSCAPE CME AUTHOR

**Laurie Barclay, MD, **freelance writer and reviewer, Medscape, LLC. *Disclosure: Laurie Barclay, MD, has disclosed no relevant financial relationships*.

## AUTHORS

Disclosures: **Jan W.M. van der Linden, MD; Eveline Snelders, MSc; Greetje A. Kampinga, MD, PhD; Bart J.A. Rijnders, MD, PhD; Eva Mattsson, MD, PhD; Yvette J. Debets-Ossenkopp, MD, PhD; Ed J. Kuijper, MD, PhD; Frank H. Van Tiel, MD, PhD;** and **Willem J.G. Melchers, PhD,** have disclosed no relevant financial relationships. **Paul E. Verweij, MD,** PhD, has disclosed the following relevant financial relationships: served as an advisor or consultant for Merck & Co., Inc.; Astellas Pharma, Inc.; Gilead Sciences, Inc.; served as a speaker or a member of a speakers bureau for Merck & Co., Inc.; Gilead Sciences, Inc.; Pfizer Inc.; Cephalon, Inc.; received grants for clinical research from Merck & Co., Inc.; Gilead Sciences, Inc.; Pfizer Inc.
