# Supplementary material for: Clinical Implications of Azole Resistance in Aspergillus fumigatus, the Netherlands, 2007–2009
Source: Emerg Infect Dis. 2011 Oct;17(10):1846–54. doi: 10.3201/eid1710.110226 (PMC3311118; doi:10.3201/eid1710.110226)
Supplement: Related texts

# Research article: Clinical Implications of Azole Resistance in *Aspergillus fumigatus*, the Netherlands, 2007–2009

**Authors:** Jan W.M. van der Linden, Eveline Snelders, Greetje A. Kampinga, Bart J.A. Rijnders, Eva Mattsson, Yvette J. Debets-Ossenkopp, Kuijper, Frank H. Van Tiel, Willem J.G. Melchers, Paul E. Verweij

**Affiliations:** Author affiliations: Radboud University Nijmegen Medical Centre, Nijmegen, the Netherlands (J.W.M. van der Linden, E. Snelders, W.J.G. Melchers, P.E. Verweij);; Groningen University Medical Centre, Groningen, the Netherlands (G.A. Kampinga);; Erasmus Medical Centre, Rotterdam, the Netherlands (B.J.A. Rijnders);; Utrecht University Medical Centre, Utrecht, the Netherlands (E. Mattsson);; Vrije University Medical Centre, Amsterdam, the Netherlands (Y.J. Debets-Ossenkopp);; Leiden University Medical Centre, Leiden, the Netherlands (E.J. Kuijper);; Maastricht University Medical Centre, Maastricht, the Netherlands (F.H. van Tiel)

## Abstract

The prevalence and spread of azole resistance in clinical *Aspergillus fumigatus* isolates in the Netherlands are currently unknown. Therefore, we performed a prospective nationwide multicenter surveillance study to determine the effects of resistance on patient management strategies and public health. From June 2007 through January 2009, all clinical *Aspergillus* spp. isolates were screened for itraconazole resistance. In total, 2,062 isolates from 1,385 patients were screened; the prevalence of itraconazole resistance in *A. fumigatus* in our patient cohort was 5.3% (range 0.8%–9.5%). Patients with a hematologic or oncologic disease were more likely to harbor an azole-resistant isolate than were other patient groups (p<0.05). Most patients (64.0%) from whom a resistant isolate was identified were azole naive, and the case-fatality rate of patients with azole-resistant invasive aspergillosis was 88.0%. Our study found that multiazole resistance in *A. fumigatus* is widespread in the Netherlands and is associated with a high death rate for patients with invasive aspergillosis.

Azoles are the primary class of antifungal agents used for evidence-based treatment and prevention of *Aspergillus* spp. diseases (*1*). Azoles are the only class of mold-active agents that can be used orally, and voriconazole and posaconazole have improved the survival of patients at risk for invasive aspergillosis (*2*–*5*). However, recent reports describe the emergence of acquired resistance of *Aspergillus* spp. to azole compounds (*6*–*16*). Azole resistance may develop in patients with cavitary lung lesions, such as aspergilloma, or in patients with allergic bronchopulmonary aspergillosis who are treated with mold-active azoles, most notably, itraconazole (*7*,*17*). In these patients, resistance is most commonly observed in *Aspergillus fumigatus*, and the isolates may be resistant to only itraconazole (ITZ) or exhibit a multi-azole– or pan-azole–resistant phenotype. The phenotype depends on the underlying resistance mechanism, which commonly involves point mutations in the *cyp51A-*gene, the target for antifungal azoles (*6*–*8*,*10*,*11*,*17*–*21*). A wide range of mutations was found in azole-resistant *Aspergillus* spp. isolates that were cultured from clinical samples from patients treated with azoles, and individual azole-resistant *Aspergillus* spp. colonies harbored different resistance mechanisms (*7*).

In the Netherlands, a highly dominant resistance mechanism was found in isolates from epidemiologically-unrelated patients (*6*,*9*). The dominant resistance mechanism consisted of a substitution of leucine for histidine at codon 98 of the *cyp51A-*gene in combination with a 34-bp tandem repeat in the promoter region of this gene (TR/L98H) (*6*,*11*). The corresponding phenotype showed resistance to itraconazole and intermediate susceptibility or resistance to voriconazole, posaconazole, or both (*6*,*20*). TR/L98H isolates were recovered from azole-treated and azole-naive patients with *Aspergillus* spp. diseases (*6*,*9*,*10*,*22*–*24*). Azole resistance may be associated with a high probability of azole treatment failure, a possibility that is supported by preclinical evidence (*7*,*9*,*22*–*26*).

On the basis of the above-mentioned observations, we hypothesized that resistance might have emerged as a consequence of exposure of *A. fumigatus* to azole fungicides in the environment (*27*). Preliminary studies indeed showed that *A. fumigatus* isolates harboring TR/L98H could be recovered from the environment (*28*,*29*). Furthermore, TR/L98H isolates were cross-resistant to certain azole fungicides (*27*,*28*).

The spread and prevalence of azole resistance in clinical *A. fumigatus* isolates are unknown. Various studies in the Netherlands that have analyzed *Aspergillus* spp. culture collections indicate that the prevalence ranges from 1.8% to 12.8% (*6*,*30*,*31*). Obtaining insight into the spread of azole resistance is essential for determining the implications of resistance for patient management strategies and public health. We therefore conducted a prospective surveillance study in 7 university medical centers in the Netherlands.

## Methods

### Study Design

All *Aspergillus* spp. isolates cultured from clinical samples that were processed in the 7 university medical microbiology laboratories were routinely screened for the presence of azole resistance, irrespective of the clinical relevance of the culture result. The University Medical Centers are located in 7 different cities in the Netherlands, and clinical isolates were screened during a 20-month period (June 2007–January 2009). Patient data were recorded in a Web-based database. An online questionnaire was completed for every collected isolate. The questionnaire included questions about isolate characteristics (species identification and date of isolation) and patient characteristics (age, sex, and underlying disease).

### Screening ITZ Agar Slants

*Aspergillus* spp. colonies that grew in primary cultures were subcultured on Sabouraud agar slants supplemented with 4 mg/L of ITZ and incubated at 35°C–37°C. The colonies’ ability to grow on the ITZ agar slants was assessed after 48 hours of incubation. For every isolate that was able to grow on the ITZ agar slants (ITZ positive), the primary culture isolate was sent to the Radboud University Nijmegen Medical Center for further analysis. Isolates that failed to grow on the ITZ agar slants (ITZ negative) were not analyzed. However, for all isolates that were screened, the online questionnaire was completed. At the screening sites, the *Aspergillus* spp. isolates were identified to species level by conventional methods, i.e., thermotolerance and macroscopic and microscopic assessment of culture morphologic features.

### Proficiency Testing

The ITZ agar slants were prepared at the Radboud University Nijmegen Medical Center and distributed to the other medical microbiology laboratories. Each center was provided with a protocol and a set of 6 *A. fumigatus* isolates. Four isolates were resistant to ITZ (MIC >16 mg/L), while 2 were susceptible (ITZ MICs 0.125 and 0.25 mg/L). The centers were blinded for the resistance profiles and were asked to determine the ability of the isolates to grow on the ITZ agar slants.

### Analysis of ITZ-positive Isolates

Every ITZ-positive isolate was analyzed for certain phenotypic and genotypic features. The phenotypic analysis included the morphologic features of the isolate and susceptibility testing according to established standards (*32*) by using a broth microdilution format. MICs were determined for ITZ, voriconazole, and posaconazole. Genotypic analyses were performed by complete sequencing of the *cyp51A* gene by using the reference sequence of strain no. AF338659 from GenBank.

For the confirmed ITZ-positive isolates, additional patient data that included azole exposure <3 months before the date of isolation of the resistant isolate and the presence of *Aspergillus* spp. disease were requested from the treating physician. Patients with cancer and invasive aspergillosis were classified according to the revised European Organization for Research and Treatment of Cancer/Mycoses Study Group definitions (*33*). In addition, patient data were collected on treatment and outcome 3 months after diagnosis.

### Statistical Analysis

Statistical analyses were performed by using SPSS version 17.0 (IBM, Somers, NY, USA). Analyses consisted of χ^2^ tests and a calculation of the κ coefficient.

## Results

### Proficiency Testing

Four of 6 control *A. fumigatus* isolates were resistant to ITZ and expected to grow on the ITZ agar slants, while the remaining 2 isolates were ITZ susceptible and should not grow on the ITZ agar slants. The 4 isolates with the azole-resistant phenotype harbored the TR/L98H resistance mechanism. For 3 ITZ-positive and the 2 ITZ-negative isolates, 100% agreement was found between the 7 centers. For 1 ITZ-positive isolate, the assessment of growth on the ITZ agar slant was incorrect from 3 centers; this isolate was a slowly sporulating *A. fumigatus* isolate. The κ coefficient calculated to assess the reproducibility of the method was 0.81 (*34*).

### Mycology

During June 2007 through January 2009, we screened 2,062 *Aspergillus* spp. isolates from 1,385 patients for azole resistance using the ITZ agar slants. Most isolates were identified as *A. fumigatus* (1,792/2,062 [86.9%]) (Table 1). For 50 (2.5%) *Aspergillus* spp. isolates, species identification was not done. Most *Aspergillus* spp. isolates were isolated from respiratory samples; 1,461 of 2,062 (70.9%) were from sputum cultures, and 60 (2.9%) were from cultures derived from clinical specimens obtained from sterile sites (i.e., tissue specimens obtained through invasive procedures or at autopsy) (Table 1).

**Table 1:** Characteristics of screened susceptible and resistant isolates of *Aspergillus* spp., the Netherlands, 2007–2009*

Source and species	No. (%) susceptible, n = 1,978	No. (%) resistant, n = 84
Specimen source		
Sputum	1,397 (70.6)	64 (76.2)
Ear swab	176 (8.9)	3 (3.6)
BAL fluid	97 (4.9)	6 (7.1)
Bronchus secretion	66 (3.3)	2 (2.4)
Throat/nasal swab	66 (3.3)	1 (1.2)
Tissue	55 (2.8)	5 (6.0)
Skin swab/nail	38 (1.9)	1 (1.2)
Mouth wash	26 (1.3)	1 (1.2)
Pus/wound swab	16 (0.8)	1 (1.2)
Bronchial wash	11 (0.6)	0
Feces	8 (0.4)	0
Unknown	22 (1.1)	0
		
		
Species		
* A. fumigatus*	1,710 (86.5)	82 (97.6)
* A. flavus*	98 (5.0)	0
* A. niger*	52 (2.6)	2 (2.4)
* A. terreus*	35 (1.8)	0
* A. nidulans*	14 (0.7)	0
* A. versicolor*	13 (0.7)	0
* A. glaucus*	6 (0.3)	0
Unknown	50 (2.5)	0

*BAL, bronchoalveolar lavage.

Ninety isolates were able to grow on the ITZ agar slants and were sent to the Radboud University Nijmegen Medical Center for further analyses. In vitro susceptibility testing showed that for 84 (93.3%) of 90 ITZ-positive isolates, the MIC of ITZ was >2 mg/L, which was considered resistant (*20*). Most ITZ-positive isolates also exhibited non–wild-type susceptibility profiles to voriconazole and posaconazole. A resistant phenotype for voriconazole (>2 mg/L) and posaconazole (>0.5 mg/L) was observed in 67/84 (79.8%) and 14/84 (16.7%) of ITZ-positive isolates, respectively. An intermediate susceptibility profile (2 mg/L for voriconazole and 0.5 mg/L for posaconazole) was observed in 12/84 (14.3%) and 44/84 (52.4%) of ITZ-positive isolates, respectively (Table 2) (*20*).

**Table 2:** Characteristics of itraconazole-positive *Aspergillus fumigatus* isolates, the Netherlands, 2007–2009*

No. isolates	Mutations in the *Cyp51A *gene	Median MIC, mg/L (range)		
		Itraconazole	Voriconazole	Posaconazole
74	TR/L98H†	>16 (16–>16)	8 (1–16)	0.5 (0.25–2)
1	G54W†	>16	0.5	>16
1	P216L†	16	2	0.5
1	F219I†	>16	0.25	0.25
1	Series‡	>16	>16	1
4	None	>16 (16–>16)	4 (0.5–4)	0.2 (0.125–1)

*In vitro susceptibility testing was performed according to the Clinical and Laboratory Standards Institute M38A2 method (*32*). †Mutations that have previously been shown to be associated with azole resistance in *A. fumigatus* (*7*,*20*,*21*). ‡Series of mutations in *Cyp51A*-gene: F46Y, G89G, M172V, N248T, D255E, L358L, E427K, and C454C (*8*).

Sequence-based identification classified 82 ITZ-positive isolates as *A. fumigatus* and 2 as *A. niger.* Sequencing of the *cyp51A* gene showed a substitution of leucine for histidine at codon 98 in combination with a 34-bp tandem repeat in the gene promoter in 74 (90.2%) of 82 resistant *A. fumigatus* isolates (Table 2). Other *cyp51A* mutations were found in 3 isolates (substitutions G54W, P216L, and F219I) (Table 2) (*7*,*8*,*20*,*21*). In 1 isolate, a series of mutations was found, and in the remaining 4 azole-resistant *A. fumigatus* isolates (4.9%), no mutations were found in the *cyp51A* gene (*8*). Because *A. niger* has no known resistance mechanisms, the 2 azole-resistant *A. niger* isolates were not further analyzed.

### Prevalence of Azole Resistance

A median of 100 isolates were screened each month, with a range of 78–140 isolates per month. The total number of screened clinical isolates per center ranged from 130 to 449 and were recovered from 84 to 238 patients. The collection of isolates over the study period and the distribution of the recovery of resistant isolates are shown in Figure 1. Overall, 82 (4.6%) of 1,792 screened *A. fumigatus* isolates were azole resistant. The per-patient analysis showed a prevalence of 5.3%. Figure 2 shows the prevalence of resistance in *A. fumigatus* per center. The prevalence of azole resistance in *A. niger* was 3.8%. No seasonal variation was observed.

**Figure 1:** Number of screened *Aspergillus* spp. isolates per month (bars) and prevalence (%) of azole resistance (line), the Netherlands, 2007–2009. ITZ, itraconazole.

**Figure 2:** Prevalence (%) of azole-resistant *Aspergillus fumigatus* infections in university medical centers, the Netherlands, 2007–2009.

### Patient Characteristics

The screened *A. fumigatus* isolates were cultured from 1,192 patients, while other *Aspergillus* spp. isolates were recovered from 193 patients. Among the patients who harbored *A. fumigatus* isolates, the predominant underlying diseases were cystic fibrosis (382/1,192 [32.1%]) and other pulmonary diseases (265/1,192 [22.2%]) Almost 12% (138/1,192) of the *A. fumigatus* isolates were from patients who had hematologic/oncologic diseases. The distribution of underlying diseases is shown in Table 3.

**Table 3:** Underlying diseases of patients from whom azole-susceptible and -resistant *Aspergillus fumigatus* isolates were recovered, the Netherlands, 2007–2009

Underlying condition	No. (%) patients with susceptible isolates, n = 1,129	No. (%) patients with resistant isolates, n = 63	p value*
Cystic fibrosis	365 (32.3)	17 (27.0)	0.38
Pulmonary disease, excluding cystic fibrosis	251 (22.2)	14 (22.2)	1.00
Hematologic/oncologic disease	125 (11.1)	13 (20.6)	0.02
Otorhinolaryngologic disease	63 (5.6)	3 (4.8)	0.78
Internal disease	85 (7.5)	8 (12.7)	0.14
Solid organ transplantation	26 (2.3)	2 (3.2)	0.66
Intensive care unit patient	31 (2.7)	2 (3.2)	0.84
Cardiac disease	9 (0.8)	1 (1.6)	0.50
Chronic granulomatous disease	5 (0.4)	0	0.60
Postoperative condition	23 (2.0)	1 (1.6)	0.81
Neurologic disease	2 (0.2)	0	0.74
Disease in children, not specified	4 (0.4)	0	0.64
Dermatologic disease	6 (0.5)	1 (1.6)	0.29
Other	91 (8.1)	1 (1.6)	
Unknown	43 (3.8)	0	

*χ^2^ test.

### Characteristics of Patients with Azole-Resistant Isolates

The 82 confirmed ITZ-resistant *A. fumigatus* isolates were recovered from 63 patients. The patients’ ages ranged from 1 to 85 years, with a mean age of 48.9 years. The sex distribution was equal.

Overall, the distribution of underlying diseases in patients with an ITZ-resistant isolate was similar to that observed in the group with ITZ-susceptible isolates, with 1 exception. In the group of patients with ITZ-resistant isolates, significantly more patients had hematologic/oncologic diseases than in the group that harbored ITZ-susceptible isolates (13/63 [20.6%] and 125/1,129 [11.1%], respectively; p<0.05; Table 3).

*Aspergillus* disease caused by an ITZ-resistant isolate was diagnosed in 23 patients (36%). Invasive aspergillosis was diagnosed in 8 patients (13%), 6 with confirmed and 2 with probable cases (Table 4). Four of these patients were azole naive, and 1 patient had been previously treated with fluconazole, which has no activity against *Aspergillus* species. All *A. fumigatus* isolates from patients with azole-resistant invasive aspergillosis harbored the TR/L98H resistance mechanism (Table 4). All 5 patients who were treated with voriconazole monotherapy died within 3 months of receiving a positive culture result. For 3 patients, voriconazole therapy was switched to another class of antifungal compounds, i.e., echinocandin, polyene, or both, but only 1 of those 3 patients survived. Overall, 7 (87.5%) of 8 patients with azole-resistant aspergillosis died within 3 months (Table 4).

**Table 4:** Characteristics of patients with azole-resistant invasive aspergillosis, the Netherlands, 2007–2009*

Patient age, y/sex	Underlying disease	Disease	No. positive cultures†	Resistance mechanism	VCZ MIC, mg/L	Prior azole treatment (duration)‡	Treatment§	Outcome at 12 wk
66/M	Lung carcinoma	Proven pulmonary aspergillosis	1	TR/L98H	4	None	VCZ	Died
59/M	Hematologic malignancy, allo-SCT, GvHD	Proven pulmonary aspergillosis	4	TR/L98H	8	VCZ (>1 mo)	VCZ	Died
54/M	Acute myeloid leukemia, relapse, allo-HSCT	Proven pulmonary aspergillosis	1	TR/L98H	8	ITZ (2–4 wk)	VCZ	Died
50/M	Non-Hodgkin lymphoma, allo-SCT, GvHD, lung cavities	Probable pulmonary aspergillosis	2	TR/L98H	16	VCZ (>1 mo)	VCZ	Died
36/F	Breast carcinoma with metastasis	Probable pulmonary aspergillosis	1	TR/L98H	1	None	VCZ	Died
13/F	Non-Hodgkin lymphoma	Proven pulmonary and CNS aspergillosis	1	TR/L98H	16	None	VCZ, CAS, AMB	Died
58/M	Liver transplantation for hepatic failure after methotrexate treatment for arteritis	Proven pulmonary and CNS aspergillosis	5	TR/L98H	2	None	AMB, VCZ	Died
60/M	Acute myeloid leukemia, allo-SCT, GvHD	Proven pulmonary and CNS aspergillosis	3	TR/L98H	4	FCZ (1–2 wk)	VCZ, CAS, AMB, POS	Survived

*VCZ, voriconazole; allo-SCT, allogeneic hematopoietic stem cell transplantation; GvHD, graft-versus-host disease; HSCT, hematopoietic stem cell transplantation; ITZ, itraconazole; CNS, central nervous system; CAS, caspofungin; AMB, amphotericin B; FCZ, fluconazole; POS, posaconazole. † All cultures were *Aspergillus fumigatus.* ‡Azole treatment <12 wk before the first culturing of an azole-resistant isolate. §Azole treatment after first culturing of resistant isolate.

Noninvasive *Aspergillus* spp. disease with an ITZ-resistant isolate was diagnosed in 15 patients. Five patients had cystic fibrosis and allergic bronchopulmonary aspergillosis. Six patients had an aspergilloma; of these patients, 2 were immunocompromised, 1 because of AIDS and 1 because of Job syndrome. Three patients sought treatment for otomycosis; 1 patient had *Aspergillus* spp. sinusitis. Data on previous exposure to azoles were available for 14 patients with noninvasive *Aspergillus* spp. disease; and 9 (64.2%) of these patients were azole naive. The TR/L98H mutation was the dominant resistance mechanism, because it was present in 12 (80%) of 15 isolates. Overall, 14 (64.3%) of 22 patients with azole-resistant *Aspergillus* spp. disease and known status of azole exposure were azole naive at the time the resistant isolate was cultured.

## Discussion

Azole resistance is widespread in clinical *A. fumigatus* isolates in the Netherlands, and resistant isolates were found at all participating university medical centers. The overall prevalence was 5.3%, which is in a similar range as observed in the fungus culture collection of the Radboud University Nijmegen Medical Center (*6*). However, the prevalence varied widely between the centers, with 1 center showing azole resistance in 10 (9.5%) of 105 patients with a positive *Aspergillus* spp. culture. In a previous study, we reported a resistance prevalence of 12.8% among *A. fumigatus* isolates sent to our department by other hospitals in the Netherlands (*6*). This higher resistance rate may have been due to a different selection of isolates. In this study, all *Aspergillus* spp. isolates cultured in the participating laboratories were screened for resistance, irrespective of their clinical relevance, while in our previous study the isolates that were sent to Nijmegen were from patients with *Aspergillus* spp. diseases for whom antifungal therapy was not successful (*6*).

Two other studies have investigated the prevalence of azole resistance in *Aspergillus* spp. in the Netherlands. One study investigated a collection of 170 clinical *A. fumigatus* isolates recovered from 114 patients from 21 different medical centers in 1945–1998 (*30*). Three (1.8%) isolates were resistant to ITZ and were later found to harbor the TR/L98H resistance mechanism. These were the first TR/L98H isolates recovered in the Netherlands (*6*). Another study investigated the prevalence of azole-resistant *Aspergillus* spp. isolates in the Netherlands by using a collection of 209 unselected clinical *A. fumigatus* isolates obtained over a 6-month period in 2005 (*31*). Only 4 azole-resistant isolates were found, which corresponds with a prevalence of 1.9% (*31*). The low prevalence may be because of the methods used. We used ITZ-containing agar-slants to select for resistant isolates, which enables detection of resistant colonies even in the presence of multiple azole-susceptible colonies. Although Klaassen et al. (*31*) did not explain how isolates were obtained, azole-resistant colonies could have been missed if regular culture media were used (*22*,*31*). A *κ *coefficient of 0.81 indicates that screening for azole resistance by using agar supplemented with ITZ is a highly reproducible method and appears to be a feasible approach for screening for azole resistance and for surveillance studies (*34*).

Most (74/82 [90.2%]) of the azole-resistant isolates in our study were found to possess the TR/L98H mutation in the *cyp51A*-gene, which is a similar proportion as reported previously (*6*). Previous studies have suggested that the probability of 2 genomic changes developing during azole therapy is highly unlikely and that exposure of *A. fumigatus* to azoles in the environment may be a possible route of resistance development (*6*,*27*,*28*). *A. fumigatus* isolates harboring the TR/L98H resistance mechanism were recovered from the environment and were genetically related to TR/L98H-containing clinical isolates (*28*). This mode of resistance development implies that previous exposure of patients to azole compounds is not a prerequisite. Indeed, our study shows that 64% of patients with an azole-resistant isolate have no history of previous azole exposure during the 3 months before culturing the isolate. This finding supports the proposed environmental route of resistance development.

The prevalence and spread of TR/L98H in *A. fumigatus* in other countries are largely unknown. A recent study by Mortensen et al. showed that 8% of environmental *A. fumigatus* isolates in Denmark harbored the TR/L98H resistance mechanism, which indicates that TR/L98H may be spreading (*29*). Because in vitro susceptibility testing of *A. fumigatus* is not routinely performed in most laboratories, international surveillance studies are warranted.

Most TR/L98H isolates were resistant to voriconazole (79%), but a broad range of MICs was observed that varied from 1 mg/L to 16 mg/L. Reasons for this variation remain unclear. TR/L98H isolates may have developed additional mutations that confer voriconazole resistance but that are not associated with the *cyp51A* gene. Another possibility is that the tandem repeat, which serves as a transcriptional enhancer, causes varying levels of up-regulation of the *cyp51A* gene. This up-regulation may result in differences in the voriconazole phenotype. More research is needed to gain insight into this phenomenon.

The outcome for patients with azole-resistant invasive aspergillosis was dismal, with a mortality rate of 88%. None of the patients who were treated with voriconazole monotherapy were alive at 3 months; of 3 patients whose treatment was switched to another class of antifungal drugs, only 1 survived. A critical issue for future study is whether azole resistance is a determinant of the poor clinical outcome of patients with azole-resistant aspergillosis. A previously reported case series of patients with primarily noninvasive *Aspergillus* spp. disease indicated that azole resistance was associated with treatment failure (*7*). However, establishing a relation between resistance and treatment outcome in invasive aspergillosis is complex because of the multiple factors that contribute to treatment outcome, including underlying disease, drug exposure, timing and accuracy of diagnosis, and timing of antifungal therapy. Furthermore, data are lacking on the effects of in vitro susceptibility patterns of *Aspergillus* spp. isolates on treatment outcomes. This relationship was recently described by using *Aspergillus* spp. isolates recovered from patients enrolled in the Transplant-Associated Infection Surveillance Network (*35*). The 12-week mortality rate for patients with culture-positive confirmed or probable invasive aspergillosis was 53% (*35*). For patients with azole-susceptible *A. fumigatus* aspergillosis treated with voriconazole, the proportion of deaths was 48% (J.W. Baddley, pers. comm.).

In a study by Herbrecht et al. (*2*), a subset of 51 modified-intent-to-treat patients with definite (43 patients) or probable (8 patients) invasive aspergillosis had a positive culture with *A. fumigatus* with a wild-type susceptibility. The all-cause proportion of deaths in this group at 12 weeks was 39% (P. Troke, pers. comm.). Both these analyses show a lower death rate than observed in our study, which indicates that azole resistance may be associated with a poor outcome. The median MIC of voriconazole in the 8 patients with azole-resistant invasive aspergillosis was 4 mg/L, which is resistant (*20*). Effects of an elevated voriconazole MIC on treatment outcome were supported by preclinical experimental results, in which mice infected with an *A. fumigatus* isolate that had a voriconazole MIC of 2 mg/L required a 2-fold higher voriconazole dose than did mice infected with a wild-type isolate to achieve a comparable survival rate (*25*).

The low survival rate of patients whose treatment was switched from azole therapy to nonazole therapy could be due to the delay of initiation of treatment with an effective alternative drug or to the presence of cerebral disease. Overall, the number of patients with azole-resistant aspergillosis in our study was low, and additional studies are needed to confirm the relation between azole resistance and treatment failure.

Because specimens from patients with invasive aspergillosis seldom produce positive cultures, our study underestimates the number of azole-resistant cases. The diagnosis of azole resistance in culture-negative cases is a challenge because current biomarkers, such as galactomannan and 1,3-β-D-glucan, do not enable species identification, let alone in vitro susceptibility testing. Molecular tools have been shown to be able to detect mutations associated with azole resistance directly in tissue (*36*) or in respiratory specimens (*37*) but are not yet suitable for use in routine patient care. Molecular tools need to be developed that enable the rapid detection of multiple mutations, although only known mutations can be detected.

Our study shows that azole resistance in clinical *A. fumigatus* isolates is widespread in the Netherlands and that the survival rate of patients with azole-resistant invasive aspergillosis is low. The dominance of the TR/L98H resistance mechanism and the high proportion of resistant isolates recovered from azole-naive patients support an environmental route of resistance development. We believe that continued surveillance is required in the Netherlands, as well as routine in vitro susceptibility testing of *A. fumigatus* isolates obtained from patients with *Aspergillus* spp. disease. International surveillance programs are also warranted to estimate the size of the emerging problem of azole resistance. Furthermore, the first-line therapy of patients with invasive aspergillosis should be reconsidered, at least in those centers with a high prevalence of azole resistance.

## References

[1] Walsh TJ, Anaissie EJ, Denning DW, Herbrecht R, Kontoyiannis DP, Marr KA, Treatment of aspergillosis: clinical practice guidelines of the Infectious Diseases Society of America. Clin Infect Dis. 2008;46:327–60. 10.1086/52525818177225

[2] Herbrecht R, Denning DW, Patterson TF, Bennett JE, Greene RE, Oestmann JW, Voriconazole versus amphotericin B for primary therapy of invasive aspergillosis. N Engl J Med. 2002;347:408–15. 10.1056/NEJMoa02019112167683

[3] Walsh TJ, Pappas P, Winston DJ, Lazarus HM, Petersen F, Raffalli J, Voriconazole compared with liposomal amphotericin B for empirical antifungal therapy in patients with neutropenia and persistent fever. N Engl J Med. 2002;346:225–34. 10.1056/NEJM20020124346040311807146

[4] Schwartz S, Ruhnke M, Ribaud P, Corey L, Driscoll T, Cornely OA, Improved outcome in central nervous system aspergillosis, using voriconazole treatment. Blood. 2005;106:2641–5. 10.1182/blood-2005-02-073315998833

[5] Slobbe L, Polinder S, Doorduijn JK, Lugtenburg PJ, el Barzouhi A, Steyerberg EW, Outcome and medical costs of patients with invasive aspergillosis and acute myelogenous leukemia-myelodysplastic syndrome treated with intensive chemotherapy: an observational study. Clin Infect Dis. 2008;47:1507–12. 10.1086/59153118990068

[6] Snelders E, van der Lee HAL, Kuijpers J, Rijs AJMM, Varga J, Samson RA, Emergence of azole resistance in *Aspergillus fumigatus* and spread of a single resistance mechanism. PLoS Med. 2008;5:e219. 10.1371/journal.pmed.005021918998768PMC2581623

[7] Howard SJ, Cerar D, Anderson MJ, Albarrag A, Fisher MC, Pasqualotto AC, Frequency and evolution of azole resistance in *Aspergillus fumigatus* associated with treatment failure. Emerg Infect Dis. 2009;15:1068–76. 10.3201/eid1507.09004319624922PMC2744247

[8] Bueid A, Howard SJ, Moore CB, Richardson MD, Harrison E, Bowyer P, Azole antifungal resistance in *Aspergillus fumigatus*: 2008 and 2009. J Antimicrob Chemother. 2010;65:2116–8. 10.1093/jac/dkq27920729241

[9] Warris A, Weemaes CM, Verweij PE. Multidrug resistance in *Aspergillus fumigatus.* N Engl J Med. 2002;347:2173–4. 10.1056/NEJM20021226347261812501236

[10] Verweij PE, Mellado E, Melchers WJ. Multiple-triazole-resistant aspergillosis. N Engl J Med. 2007;356:1481–3. 10.1056/NEJMc06172017409336

[11] Mellado E, Garcia-Effron G, Alcázar-Fuoli L, Melchers WJ, Verweij PE, Cuenca-Estrella M, A new *Aspergillus fumigatus* resistance mechanism conferring in vitro cross-resistance to azole antifungals involves a combination of *cyp51A* alterations. Antimicrob Agents Chemother. 2007;51:1897–904. 10.1128/AAC.01092-0617371828PMC1891382

[12] Lagrou K, De Vleeschouwer J, Meerseman W, Dupont L, Verleden G, Melchers WJG, Triazole resistance among 229 clinical *Aspergillus fumigatus* isolates. Presented at: 3rd Advances Against Aspergillosis Conference; Miami, Florida, USA; January 16–19, 2008. Abstract 33.

[13] Arendrup MC, Perkhofer S, Howard SJ, Garcia-Effron G, Vishukumar A, Perlin D, Establishing in vitro–in vivo correlations for *Aspergillus fumigatus*: the challenge of azoles versus echinocandins. Antimicrob Agents Chemother. 2008;52:3504–11. 10.1128/AAC.00190-0818644959PMC2565905

[14] Chryssanthou E. In vitro susceptibility of respiratory isolates of *Aspergillus* species to itraconazole and amphotericin B acquired resistance to itraconazole. Scand J Infect Dis. 1997;29:509–12. 10.3109/003655497090118649435042

[15] Dannaoui E, Borel E, Monier MF, Piens MA, Picot S, Persat F. Acquired itraconazole resistance in *Aspergillus fumigatus.* J Antimicrob Chemother. 2001;47:333–40. 10.1093/jac/47.3.33311222566

[16] Denning DW, Radford SA, Oakley KL, Hall L, Johnson EM, Warnock DW. Correlation between in-vitro susceptibility testing to itraconazole and in-vivo outcome of *Aspergillus fumigatus* infection. J Antimicrob Chemother. 1997;40:401–14. 10.1093/jac/40.3.4019338494

[17] Chen J, Li H, Li R, Bu D, Wan Z. Mutations in the *cyp51A* gene and susceptibility to itraconazole in *Aspergillus fumigatus* serially isolated from a patient with lung aspergilloma. J Antimicrob Chemother. 2005;55:31–7. 10.1093/jac/dkh50715563516

[18] Mellado E, Garcia-Effron G, Alcazar-Fuoli L, Cuenca-Estrella M, Rodriguez-Tudela JL. Substitutions at methionine 220 in the 14alpha-sterol demethylase *(Cyp51A)* of *Aspergillus fumigatus* are responsible for resistance in vitro to azole antifungal drugs. Antimicrob Agents Chemother. 2004;48:2747–50. 10.1128/AAC.48.7.2747-2750.200415215142PMC434164

[19] Diaz-Guerra TM, Mellado E, Cuenca-Estrella M, Rodriguez-Tudela JL. A point mutation in the 14alpha-sterol demethylase gene *cyp51A* contributes to itraconazole resistance in *Aspergillus fumigatus.* Antimicrob Agents Chemother. 2003;47:1120–4. 10.1128/AAC.47.3.1120-1124.200312604551PMC149335

[20] Verweij PE, Howard SJ, Melchers WJG, Denning DW. Azole resistance in *Aspergillus*: proposed nomenclature and breakpoints. Drug Resist Updat. 2009;12:141–7. 10.1016/j.drup.2009.09.00219879181

[21] Snelders E. Karawajczyk, Schaftenaar G, Verweij PE, Melchers WJ. Azole resistance profile of amino acid changes in *Aspergillus fumigatus cyp51A* based on protein homology modeling. Antimicrob Agents Chemother. 2010;54:2425–30. 10.1128/AAC.01599-0920385860PMC2876375

[22] van Leer-Buter C, Takes RP, Hebeda KM, Melchers WJ, Verweij PE. Aspergillosis–and a misleading sensitivity result. Lancet. 2007;370:102. 10.1016/S0140-6736(07)61055-117617278

[23] van der Linden JW, Jansen RR, Bresters D, Visser CE, Geerlings SE, Kuijper EJ, Azole resistant central nervous system aspergillosis. Clin Infect Dis. 2009;48:1111–3. 10.1086/59746519272019

[24] Hodiamont CJ, Dolman KM, ten Berge RJM, Melchers WJ, Verweij PE, Pajkrt D. Multiple-azole-resistant *Aspergillus fumigatus* osteomyelitis in a patient with chronic granulomatous disease successfully treated with long-term oral posaconazole and surgery. Med Mycol. 2009;47:217–20. 10.1080/1369378080254560019101840

[25] Mavridou E, Brüggemann RJ, Melchers WJ, Verweij PE, Mouton JW. Impact of *cyp51A* mutations on the pharmacokinetic and pharmacodynamic properties of voriconazole in a murine model of disseminated aspergillosis. Antimicrob Agents Chemother. 2010;54:4758–64. 10.1128/AAC.00606-1020733046PMC2976127

[26] Mavridou E, Brüggemann RJ, Melchers WJ, Mouton JW, Verweij PE. Efficacy of posaconazole against three clinical *Aspergillus fumigatus* isolates with mutations in the *cyp51A* gene. Antimicrob Agents Chemother. 2010;54:860–5. 10.1128/AAC.00931-0919917751PMC2812170

[27] Verweij PE, Snelders E, Kema GH, Mellado E, Melchers WJ. Azole resistance in *Aspergillus fumigatus*: a side-effect of environmental fungicide use? Lancet Infect Dis. 2009;9:789–95. 10.1016/S1473-3099(09)70265-819926038

[28] Snelders E, Huis in ‘t Veld RAG, Rijs AJJM, Kema GHJ, Melchers WJ, Verweij PE. Possible environmental origin of resistance of *Aspergillus fumigatus* to medical triazoles. Appl Environ Microbiol. 2009;75:4053–7. 10.1128/AEM.00231-0919376899PMC2698372

[29] Mortensen KL, Mellado E, Lass-Flörl C, Rodriguez-Tudela JL, Johansen HK, Arendrup MC. Environmental study of azole-resistant *Aspergillus fumigatus* and other aspergilli in Austria, Denmark, and Spain. Antimicrob Agents Chemother. 2010;54:4545–9. 10.1128/AAC.00692-1020805399PMC2976122

[30] Verweij PE, Te Dorsthorst DT, Rijs AJ, De Vries-Hospers HG, Meis JF. Nationwide survey of in vitro activities of itraconazole and voriconazole against clinical *Aspergillus fumigatus* isolates cultured between 1945 and 1998. J Clin Microbiol. 2002;40:2648–50. 10.1128/JCM.40.7.2648-2650.200212089298PMC120540

[31] Klaassen CH, de Valk HA, Curfs-Breuker IM, Meis JF. Novel mixed-format real-time PCR assay to detect mutations conferring resistance to triazoles in *Aspergillus fumigatus* and prevalence of multi-triazole resistance among clinical isolates in the Netherlands. J Antimicrob Chemother. 2010;65:901–5. 10.1093/jac/dkq04120167588

[32] Clinical and Laboratory Standards Institute. Reference method for broth dilution antifungal susceptibility testing of filamentous fungi; approved standard, 2nd ed. CLSI document M38–A2. Wayne (PA): The Institute; 2008.

[33] De Pauw B, Walsh TJ, Donnelly JP, Stevens DA, Edwards JE, Calandra T, Revised definitions of invasive fungal disease from the EORTC/MSG. Clin Infect Dis. 2008;46:1813–21. 10.1086/58866018462102PMC2671227

[34] Landis JR, Koch GG. The measurement of observer agreement for categorical data. Biometrics. 1977;33:159–74. 10.2307/2529310843571

[35] Baddley JW, Marr KA, Andes DR, Walsh TJ, Kauffman CA, Kontoyiannis DP, Patterns of susceptibility of *Aspergillus* isolates recovered from patients enrolled in the Transplant-Associated Infection Surveillance Network. J Clin Microbiol. 2009;47:3271–5. 10.1128/JCM.00854-0919692558PMC2756905

[36] van der Linden JW, Snelders E, Arends JP, Daenen SM, Melchers WJ, Verweij PE. Rapid diagnosis of azole-resistant aspergillosis by direct PCR using tissue specimens. J Clin Microbiol. 2010;48:1478–80. 10.1128/JCM.02221-0920107096PMC2849623

[37] Denning DW, Park S, Lass-Florl C, Fraczek MG, Kirwan M, Gore R, High-frequency triazole resistance found in nonculturable *Aspergillus fumigatus* from lungs of patients with chronic fungal disease. Clin Infect Dis. 2011;52:1123–9. 10.1093/cid/cir17921467016PMC3106268

# Research article: Clinical Implications of Azole Resistance in *Aspergillus fumigatus*, the Netherlands, 2007–2009

**DOI:** 10.3201/eid1710.110226

**Authors:** Jan W.M. van der Linden, Eveline Snelders, Greetje A. Kampinga, Bart J.A. Rijnders, Eva Mattsson, Yvette J. Debets-Ossenkopp, Kuijper, Frank H. Van Tiel, Willem J.G. Melchers, Paul E. Verweij

**Affiliations:** Author affiliations: Radboud University Nijmegen Medical Centre, Nijmegen, the Netherlands (J.W.M. van der Linden, E. Snelders, W.J.G. Melchers, P.E. Verweij);; Groningen University Medical Centre, Groningen, the Netherlands (G.A. Kampinga);; Erasmus Medical Centre, Rotterdam, the Netherlands (B.J.A. Rijnders);; Utrecht University Medical Centre, Utrecht, the Netherlands (E. Mattsson);; Vrije University Medical Centre, Amsterdam, the Netherlands (Y.J. Debets-Ossenkopp);; Leiden University Medical Centre, Leiden, the Netherlands (E.J. Kuijper);; Maastricht University Medical Centre, Maastricht, the Netherlands (F.H. van Tiel)

**Earning MEDSCAPE CME Credit**To obtain credit, you should first read the journal article. After reading the article, you should be able to answer the following, related, multiple-choice questions. To complete the questions (with a minimum 70% passing score) and earn continuing medical education (CME) credit, please go to www.medscape.org/journal/eid. Credit cannot be obtained for tests completed on paper, although you may use the worksheet below to keep a record of your answers. You must be a registered user on Medscape.org. If you are not registered on Medscape.org, please click on the New Users: Free Registration link on the left hand side of the website to register. Only one answer is correct for each question. Once you successfully answer all post-test questions you will be able to view and/or print your certificate. For questions regarding the content of this activity, contact the accredited provider, CME@medscape.net. For technical assistance, contact CME@webmd.net. American Medical Association’s Physician’s Recognition Award (AMA PRA) credits are accepted in the US as evidence of participation in CME activities. For further information on this award, please refer to http://www.ama-assn.org/ama/pub/category/2922.html. The AMA has determined that physicians not licensed in the US who participate in this CME activity are eligible for *AMA PRA Category 1 Credits*™. Through agreements that the AMA has made with agencies in some countries, AMA PRA credit may be acceptable as evidence of participation in CME activities. If you are not licensed in the US, please complete the questions online, print the certificate and present it to your national medical association for review.**Article Title: Clinical Implications of Azole Resistance in *Aspergillus fumigatus*, the Netherlands, 2007–2009MEDSCAPE CME Questions1. Which of the following statements about the prevalence of itraconazole resistance in clinical *Aspergillus fumigatus* isolates is most likely correct?**A. Overall prevalence was 5.3%B. Resistant isolates were found in half of participating university medical centersC. The range of prevalence at participating university medical centers was 0%–6%D. One quarter of azole-resistant isolates had the TR/L98H mutation in the Cyp51A gene**2. A 34-year-old white woman presents with non-Hodgkin lymphoma, pulmonary symptoms, and positive sputum culture for *A. fumigatus*. On the basis of the study by van der Linden and colleagues, which of the following statements about risk factors for development of itraconazole resistance in *A. fumigatus* isolates is most likely correct?**A. History of a hematologic/oncologic disease is not a risk factorB. Previous azole treatment is necessary for development of resistanceC. Most TR/L98H mutations are resistant to voriconazole and have a narrow range of minimum inhibitory concentrationsD. Azole resistance may develop in patients with cavitary lung lesions such as aspergilloma**3. Which of the following statements about outcomes associated with development of itraconazole resistance in *A. fumigatus* isolates is most likely to apply to the previously described patient?**A. The mortality rate of patients with azole-resistant invasive aspergillosis is ≈50%B. Two thirds of patients whose treatment was switched to another class of antifungal survivedC. None of the patients who were treated with voriconazole monotherapy were alive at 3 monthsD. This study proved that azole resistance causes poor clinical outcomes in patients with azole-resistant aspergillosis

## Activity Evaluation

**Table:** 

1. The activity supported the learning objectives.				
Strongly Disagree				Strongly Agree
1	2	3	4	5
2. The material was organized clearly for learning to occur.				
Strongly Disagree				Strongly Agree
1	2	3	4	5
3. The content learned from this activity will impact my practice.				
Strongly Disagree				Strongly Agree
1	2	3	4	5
4. The activity was presented objectively and free of commercial bias.				
Strongly Disagree				Strongly Agree
1	2	3	4	5
